# Effect of Subcutaneous Anti-CD20 Antibody-Mediated B Cell Depletion on Susceptibility to Pneumocystis Infection in Mice

**DOI:** 10.1128/mSphere.01144-20

**Published:** 2021-05-05

**Authors:** Guixiang Dai, Kristin Noell, Gisbert Weckbecker, Jay K. Kolls

**Affiliations:** a Center for Translational Research in Infection and Inflammation, Tulane University School of Medicine, New Orleans, Louisiana, USA; b Novartis, Basel, Switzerland; University of Georgia

**Keywords:** B cells, CD20, *Pneumocystis*

## Abstract

Prior work has shown that parenterally administered anti-CD20 (5D2) inhibits CD4^+^ T cell priming in response to challenge with Pneumocystis murina and predisposes to pneumonia. In this study, we investigated the effect of subcutaneous anti-CD20 antibody and Pneumocystis infection. In mice with primary infection, anti-CD20 antibody treatment depleted both CD19^+^ and CD27^+^ CD19^+^ cells but not T cells in the lung at days 14 and 28 after Pneumocystis inoculation. Although anti-CD20 antibody treatment impaired fungal clearance at day 14 postinfection, fungal burden in the lungs was substantially reduced at day 28 in both depleted and control mice in the low-dose group. Subcutaneous anti-CD20 antibody treatment did not alter antigen-specific serum immunoglobulin levels in mice compared with control mice, and there were no significant differences in the numbers of lung gamma interferon-positive (IFN-γ^+^) CD4^+^, interleukin 4-positive (IL-4^+^) CD4^+^, IL-5^+^ CD4^+^, and IL-17A^+^ CD4^+^ cells between depleted and control mice after infection. In mice with secondary infection, the lung fungal burden was comparable between depleted and control mice 14 days after reinfection. Low-dose subcutaneous anti-CD20 antibody treatment may delay fungal clearance, but it did not impair the ability of the host to clear Pneumocystis infection, irrespective of primary or secondary infection.

**IMPORTANCE** Anti-CD20 antibody therapy is used for both cancer and autoimmune disease but has been shown to be associated with Pneumocystis pneumonia in humans. This study shows that low-dose subcutaneous anti-CD20 can modulate B cell populations without grossly perturbing fungal immunity against Pneumocystis lung infection.

## Abstract (precis)

Anti-CD20 antibody therapy is used for both cancer and autoimmune disease but has been shown to be associated with Pneumocystis pneumonia in humans. This study shows that low-dose subcutaneous anti-CD20 can modulate B cell populations without grossly perturbing fungal immunity against Pneumocystis lung infection.

## INTRODUCTION

B cells play critical roles in host immunity against Pneumocystis infection. This includes a critical role as class II major histocompatibility complex (MHC) antigen-presenting cells as well as antibody-secreting cells that can prevent secondary infection (1–3). Anti-CD20 antibody use in humans has been associated with Pneumocystis infection (4–7), and recently, we showed that administration of 5D2 by the intraperitoneal route resulted in significant B cell depletion and a failure to adequately prime T cells in the lung in response to Pneumocystis infection (3).

In this study, we studied the effect of subcutaneous administration of anti-CD20. We found that subcutaneous anti-CD20 antibody treatment depleted both CD19^+^ and CD27^+^ CD19^+^ cells but not T cells in the lung at days 14 and 28 after Pneumocystis inoculation. Although the anti-CD20 antibody treatment impaired fungal clearance at day 14 postinfection, fungal burden in the lungs was substantially reduced at day 28 in both low-dose depleted and control mice. There was also a modest reduction in fungal burden in the high-dose anti-CD20 group. Anti-CD20 antibody treatment did not alter antigen-specific serum immunoglobulin levels compared with control mice, and there were no significant differences in the numbers of lung gamma interferon-positive (IFN-γ^+^) CD4^+^, interleukin 4-positive (IL-4^+^) CD4^+^, IL-5^+^ CD4^+^, and IL-17A^+^ CD4^+^ cells between depleted and control mice after infection. In mice with secondary infection, the lung fungal burdens were comparable between depleted and control mice 14 days after reinfection. Low-dose subcutaneous anti-CD20 antibody treatment may delay fungal clearance, but it did not impair the ability of the host to clear Pneumocystis infection, irrespective of primary or secondary infection.

## RESULTS

### Effect of anti-CD20 on primary infection.

**FIG 1:**
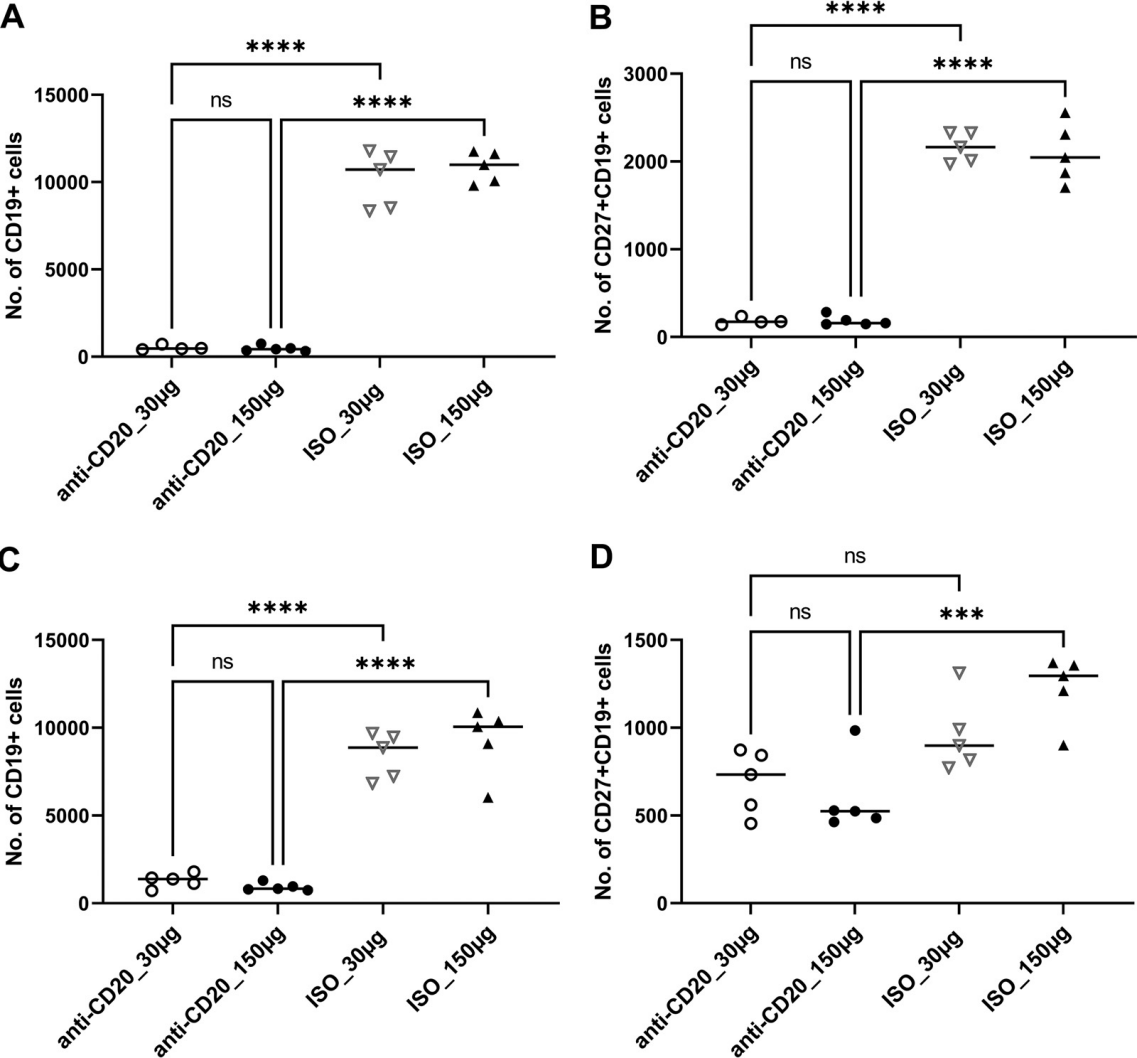
Lung CD19^+^ and CD27^+^ CD19^+^ cells at day 14 (A and B) and day 28 (C and D) postinfection. C57BL/6 mice were subcutaneously given 30 or 150 μg of anti-CD20 or isotype control antibody weekly. Three days after the first injection, all mice were inoculated with approximately 2 × 10^5^ asci of P. murina inoculum. Fourteen or 28 days postinfection, the right upper and lower lung lobes were harvested, and single cells were prepared and stimulated with *Pneumocystis* (PC) antigen for 6 h followed by staining for cell surface markers and intracellular cytokine staining. For CD19^+^ and CD27^+^ CD19^+^ cells, 100,000 cells were acquired. One-way ANOVA and Tukey’s multiple-comparison tests indicated that there were significant differences between anti-CD20 and isotype control antibody (ISO)-treated groups. Statistical significance is indicated as follows: ***, *P ≤ *0.001; ****, *P ≤ *0.0001; ns, not significant.

**FIG 2:**
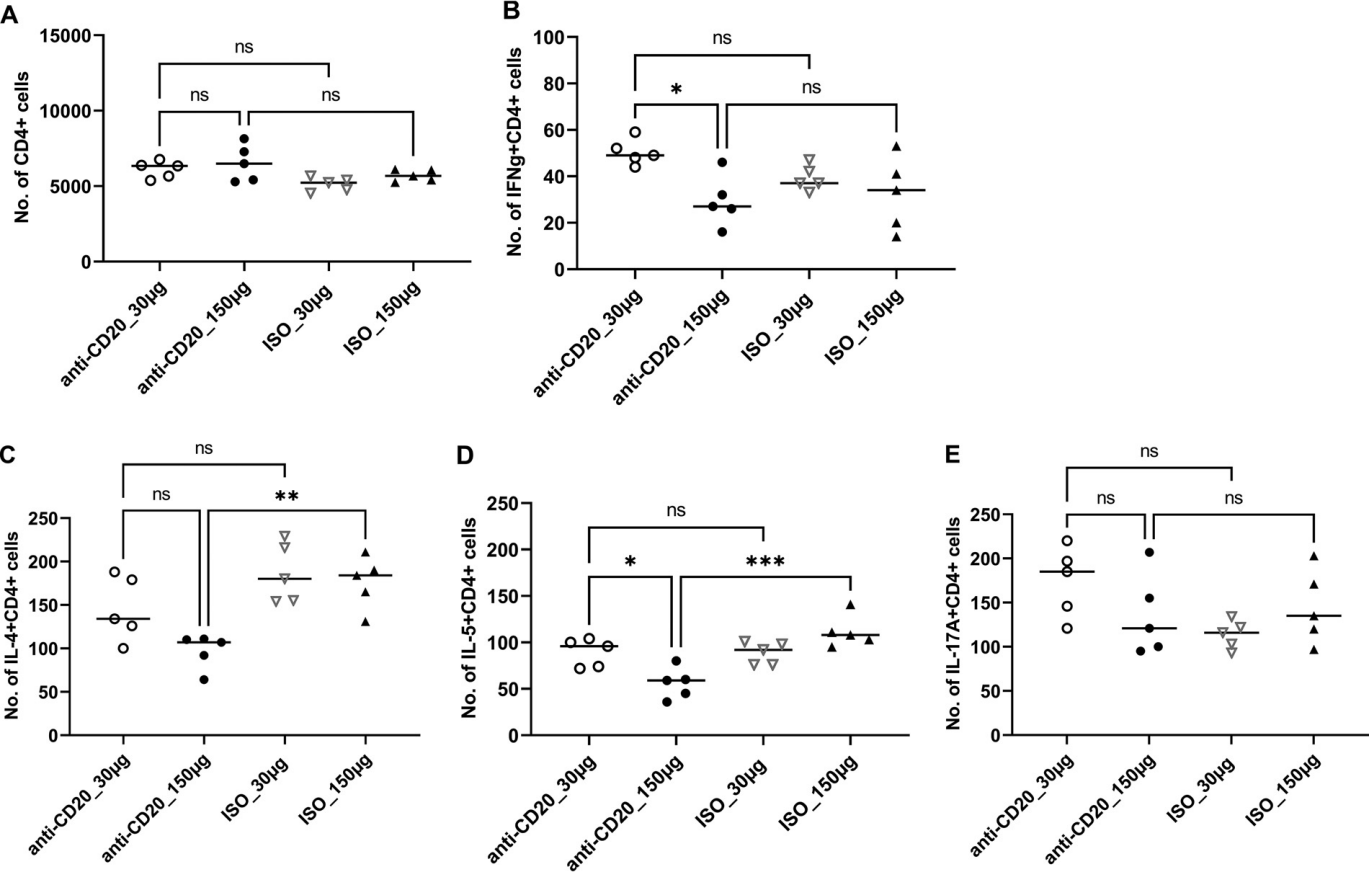
Lung CD4^+^ T cells and CD4^+^ T cell subsets at day 14 postinfection. C57BL/6 mice were subcutaneously given 30 or 150 μg of anti-CD20 or isotype control antibody weekly. Three days after the first injection, all mice were inoculated with approximately 2 × 10^5^ asci of P. murina inoculum. Fourteen days postinfection, the right upper and lower lung lobes were harvested, and single cells were prepared and stimulated with PC antigen for 6 h followed by staining for cell surface markers and intracellular cytokine staining. For CD4^+^ cells and subsets, 100,000 cells were acquired. One-way ANOVA and Tukey’s multiple-comparison tests indicated that there were significant differences for IL-4^+^ CD4^+^ and IL-5^+^ CD4^+^ subsets between anti-CD20- and ISO-treated groups at higher treatment dosages. Low (30 μg) and high (150 μg) doses of anti-CD20 treatment did not impact the CD4^+^ T cell number in panel A. Statistical significance is indicated as follows: *, *P ≤ *0.05; **, *P ≤ *0.01; ***, *P ≤ *0.001; ns, not significant.

Mice were randomized to receive low-dose (30-μg) or high-dose (150-μg) anti-CD20 or isotype control weekly starting 3 days prior to fungal inoculation. Fourteen days after fungal inoculation, lungs were harvested, and B cells were enumerated by staining with anti-CD19 and anti-CD27. Both the low dose and high dose of anti-CD20 significantly reduced CD19^+^ and CD19/CD27^+^ B cell populations in the lung at day 14 (Fig. 1A and B) and day 28 (Fig. 1C and D). Despite this, there was a minimal effect on total lung CD4^+^ T cell populations (Fig. 2). In fact, total lung CD4^+^ T cells were slightly increased in the anti-CD20 group which was statistically significant in the high-dose anti-CD20 group (Fig. 2A). High-dose anti-CD20 was associated with marginally reduced Th1 (Fig. 2B) but significantly reduced Th2 cells (Fig. 2C and D), whereas there were no differences observed in Th17 cells (Fig. 2E).

**FIG 3:**
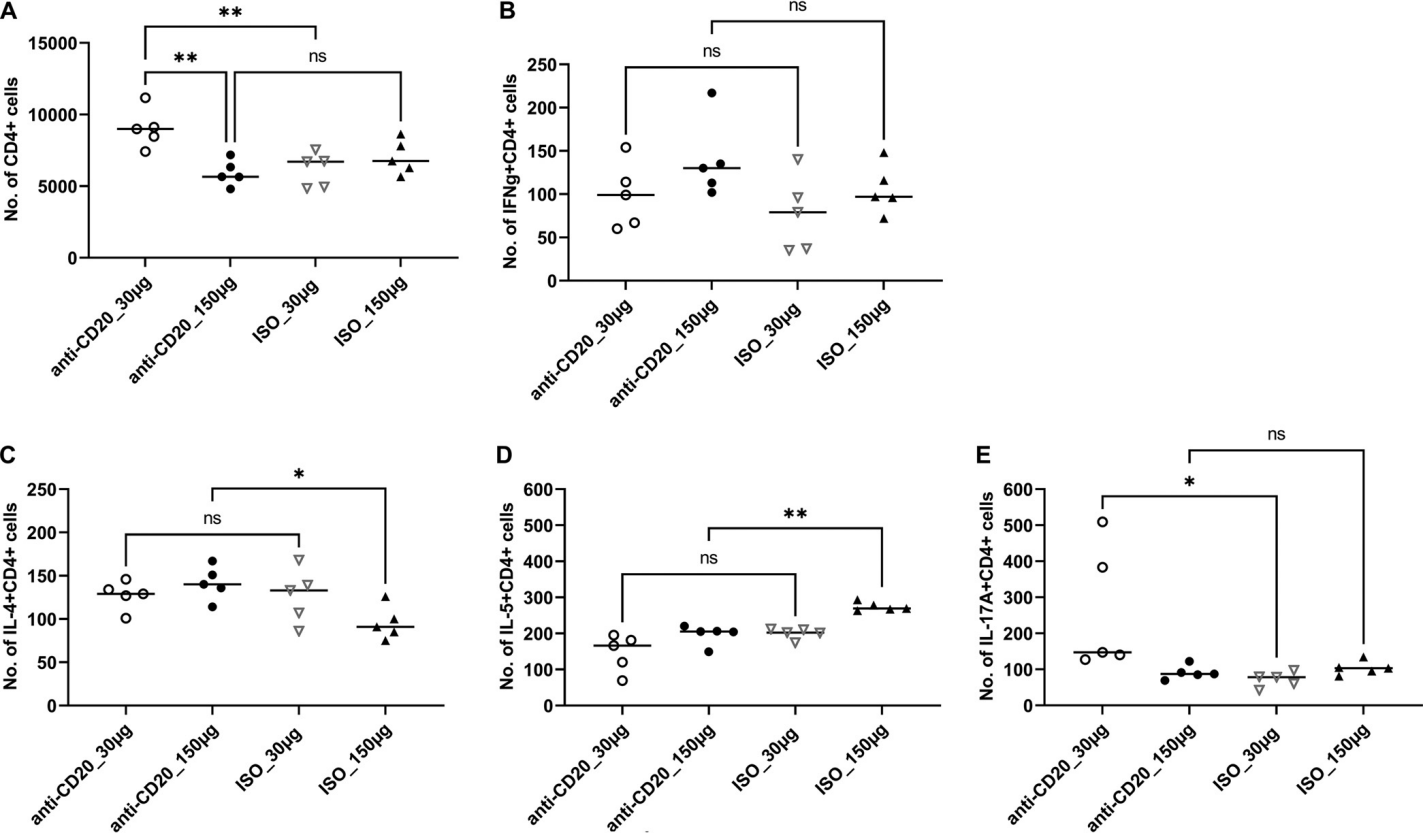
Lung CD4^+^ T cells and CD4^+^ T cell subsets at day 28 postinfection. C57BL/6 mice were subcutaneously given 30 or 150 μg of anti-CD20 or isotype control antibody weekly. Three days after the first injection, all mice were inoculated with approximately 2 × 10^5^ asci of P. murina inoculum. Twenty-eight days postinfection, the right upper and lower lung lobes were harvested, and single cells were prepared and stimulated with PC antigen for 6 h followed by staining for cell surface markers and intracellular cytokine staining. For CD4^+^ cells and subsets, 100,000 cells were acquired. One-way ANOVA and Tukey’s multiple-comparison tests indicated that there were significant differences for IL-4^+^ CD4^+^ and IL-5^+^ CD4^+^ subsets between anti-CD20- and ISO-treated groups at higher treatment dosages. There were significantly fewer CD4^+^ T cells in the higher-treatment-dosage group (*P* < 0.01) in panel A. Statistical significance is indicated as follows: *, *P ≤ *0.05; **, *P ≤ *0.01; ns, not significant.

**FIG 4:**
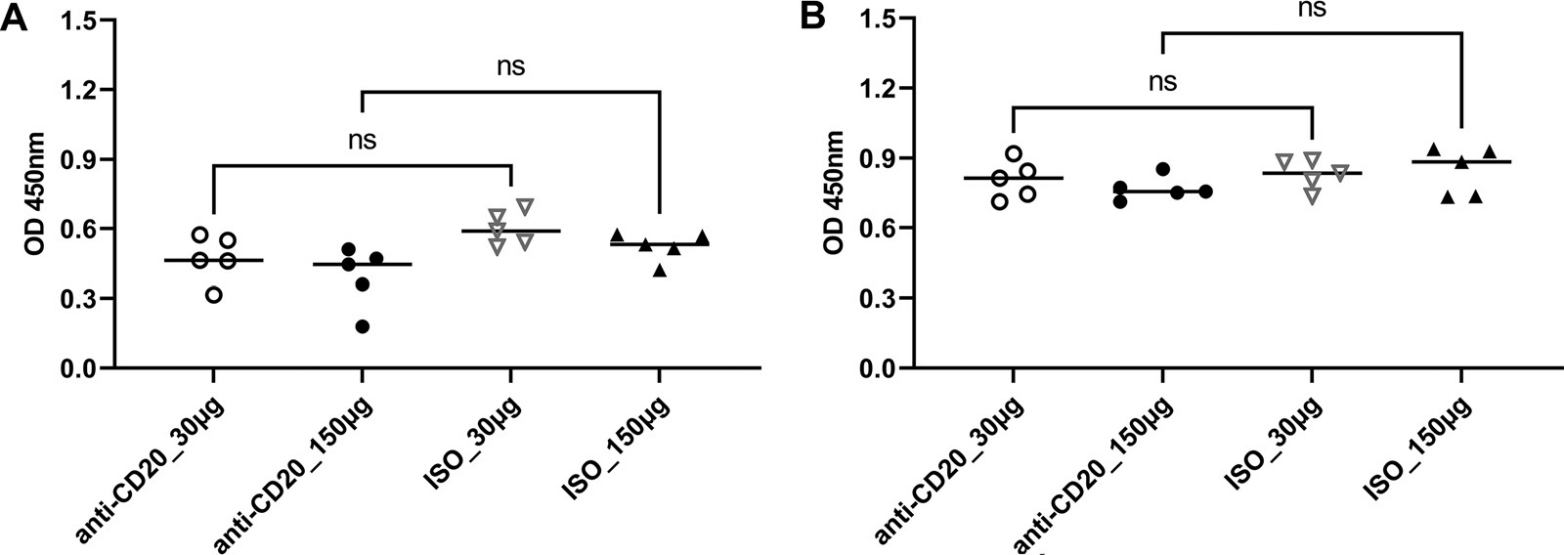
Pneumocystis-specific IgG in sera. C57BL/6 mice were subcutaneously given 30 or 150 μg of anti-CD20 or isotype control antibody weekly. Three days after the first injection, all mice were inoculated with approximately 2 × 10^5^ asci of P. murina inoculum. Fourteen and 28 days postinfection, groups of mice were sacrificed, and sera were taken for ELISA. One-way ANOVA and Tukey’s multiple-comparison tests indicated that there were no significant differences in serum IgG between anti-CD20- and ISO-treated groups at both day 14 (A) and day 28 (B). ns, not significant.

**FIG 5:**
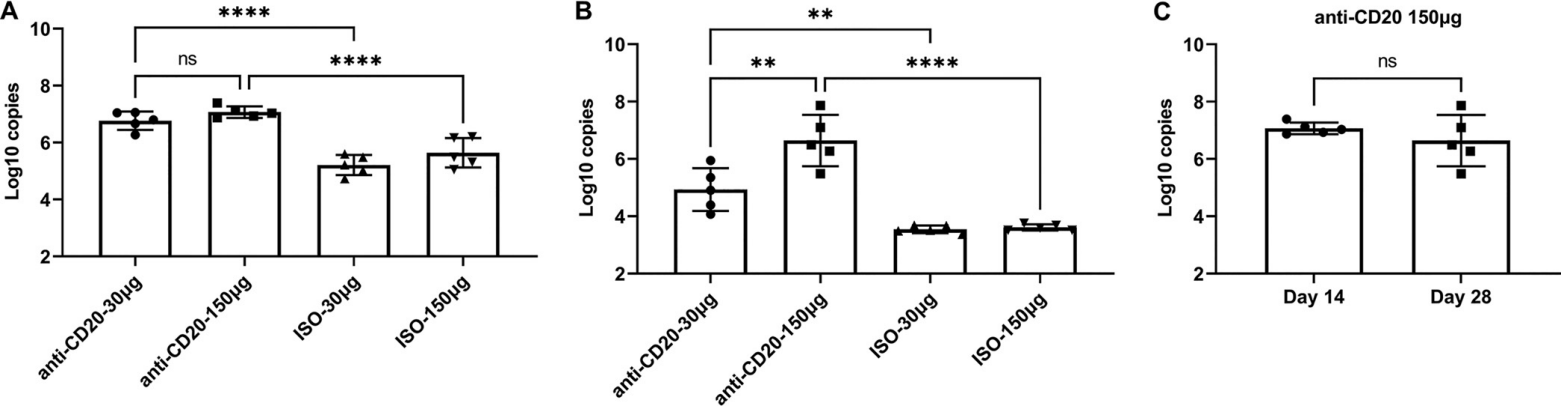
Lung fungal burdens at day 14 (A) and day 28 (B) postinfection. C57BL/6 mice were subcutaneously given 30 or 150 μg of anti-CD20 or isotype control antibody weekly. Three days after the first injection, all mice were inoculated with approximately 2 × 10^5^ asci of P. murina inoculum. Fourteen or 28 days postinfection, the right middle lung lobes were taken, and RNA was extracted for assessing lung fungal burden by real-time quantitative PCR (RT-qPCR). One-way ANOVA and Tukey’s multiple-comparison tests indicated that there were significant differences in the fungal burden between anti-CD20- and ISO-treated groups on both day 14 and day 28. There was no difference in the fugal burden between 30-μg and 150-μg treatment groups on day 14 (A), while significantly higher fungal burden was measured on day 28 in the higher-treatment-dosage group (B). For high-dose anti-CD20 treatment, the results of a two-tailed unpaired *t* test showed that there was no difference in the fungal burden between day 14 and day 28 (C). ns, not significant.

These significantly reduced Th2 responses were associated with an increase in lung fungal burden at day 14 after fungal inoculation (see Fig. 5A). By day 28, there was a modest increase in lung CD4^+^ T cells in the low-dose anti-CD20 group but no significant differences in the other three groups (Fig. 3A). There were no differences in lung IFN-γ^+^ CD4^+^ cells (Fig. 3B), while IL-4^+^ CD4^+^ and IL-5^+^ CD4^+^ subsets were altered in the high-dose groups (Fig. 3C and D), and Th17 responses were increased in the low-dose cohort (Fig. 3E). There were no differences in lung Th1 or Th2 cells (Fig. 3 and 3D), but Th17 responses were increased in the low-dose cohort (Fig. 3E). Mice treated with anti-CD20 were also able to mount significant antifungal IgG response (Fig. 4A and B) with a modest reduction at day 14 in the high-dose anti-CD20 group. However, by day 28 there were no differences in antifungal IgG between groups (see Fig. S1 in the supplemental material). Fungal burdens were modestly elevated at day 28 in the low-dose anti-CD20 group but were significantly lower than day 14 time point (Fig. 5B), consistent with intact fungal clearance in the low-dose anti-CD20-treated mice. Fungal burdens remained elevated in the high-dose anti-CD20 arm which were comparable to the fungal burdens at day 14 consistent with a lack of fungal clearance (Fig. 5C).

10.1128/mSphere.01144-20.1 FIG S1 C57BL/6 mice were subcutaneously given 30 or 150 μg of anti-CD20 or isotype control antibody from Novartis weekly. Three days after the first injection, all mice were administered 100 microliters (approximately 2 × 10^5^ asci) of P. murina inoculum by oral pharyngeal aspiration (tongue pull method). Twenty-eight days postinfection, groups of mice were sacrificed, and sera were assayed for anti-PC IgG by ELISA. Download FIG S1, PDF file, 0.2 MB. Copyright © 2021 Dai et al. 2021 Dai et al. https://creativecommons.org/licenses/by/4.0/ This content is distributed under the terms of the Creative Commons Attribution 4.0 International license.

### Effect of anti-CD20 on secondary infection.

**FIG 6:**
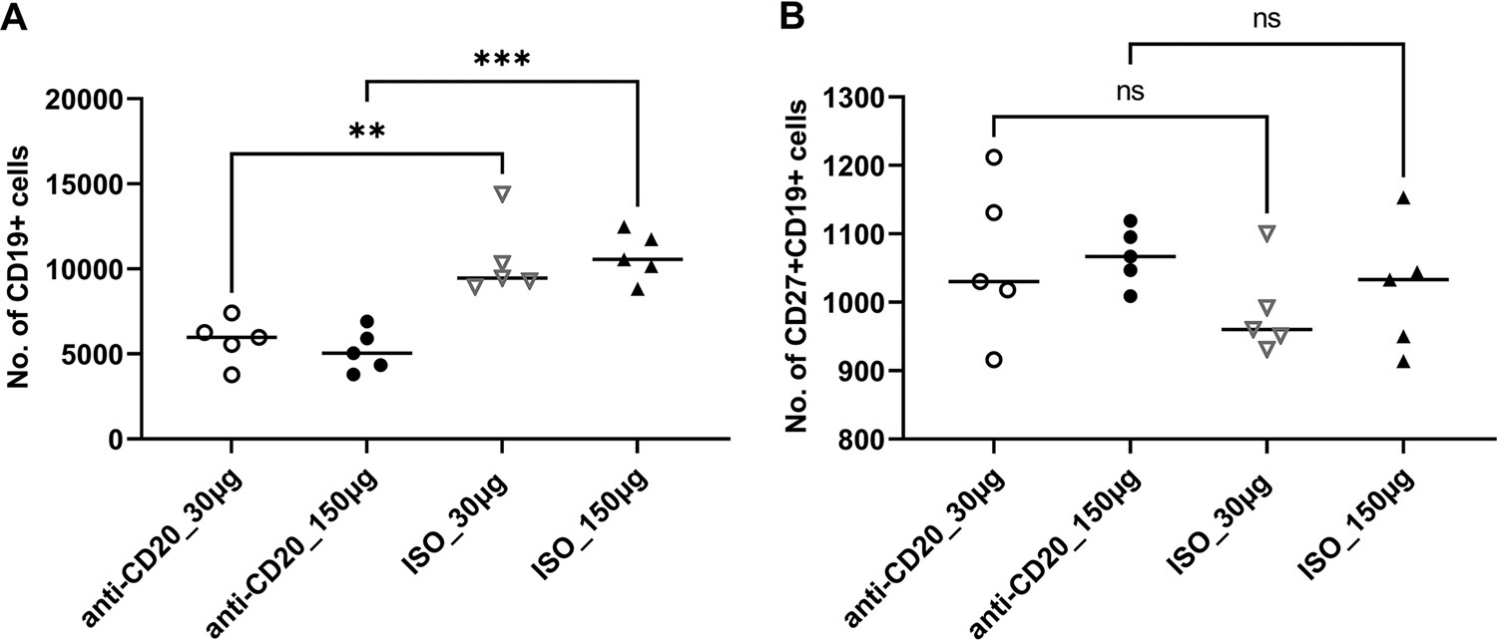
Lung CD19^+^ and CD27^+^ CD19^+^ cells in secondary PC infection. All mice were inoculated with approximately 2 × 10^5^ asci of P. murina inoculum. Six weeks later, the mice were subcutaneously given 30 or 150 μg of anti-CD20 or isotype control antibody weekly. Three days after the first injection, all mice were reinoculated with approximately 2 × 10^5^ asci of P. murina inoculum. Fourteen days postinfection, the right upper and lower lung lobes were taken, and single cells were prepared and stimulated with PC antigen for 6 h for surface markers and intracellular cytokine staining. For both CD19^+^ and CD27^+^ CD19^+^ cells, 100,000 cells were acquired. One-way ANOVA and Tukey’s multiple-comparison tests indicated that there were significant differences between anti-CD20- and ISO-treated groups for CD19^+^ cells but not for CD27^+^ CD19^+^ cells. Statistical significance is indicated as follows: **, *P ≤ *0.01; ***, *P ≤ *0.001; ns, not significant.

**FIG 7:**
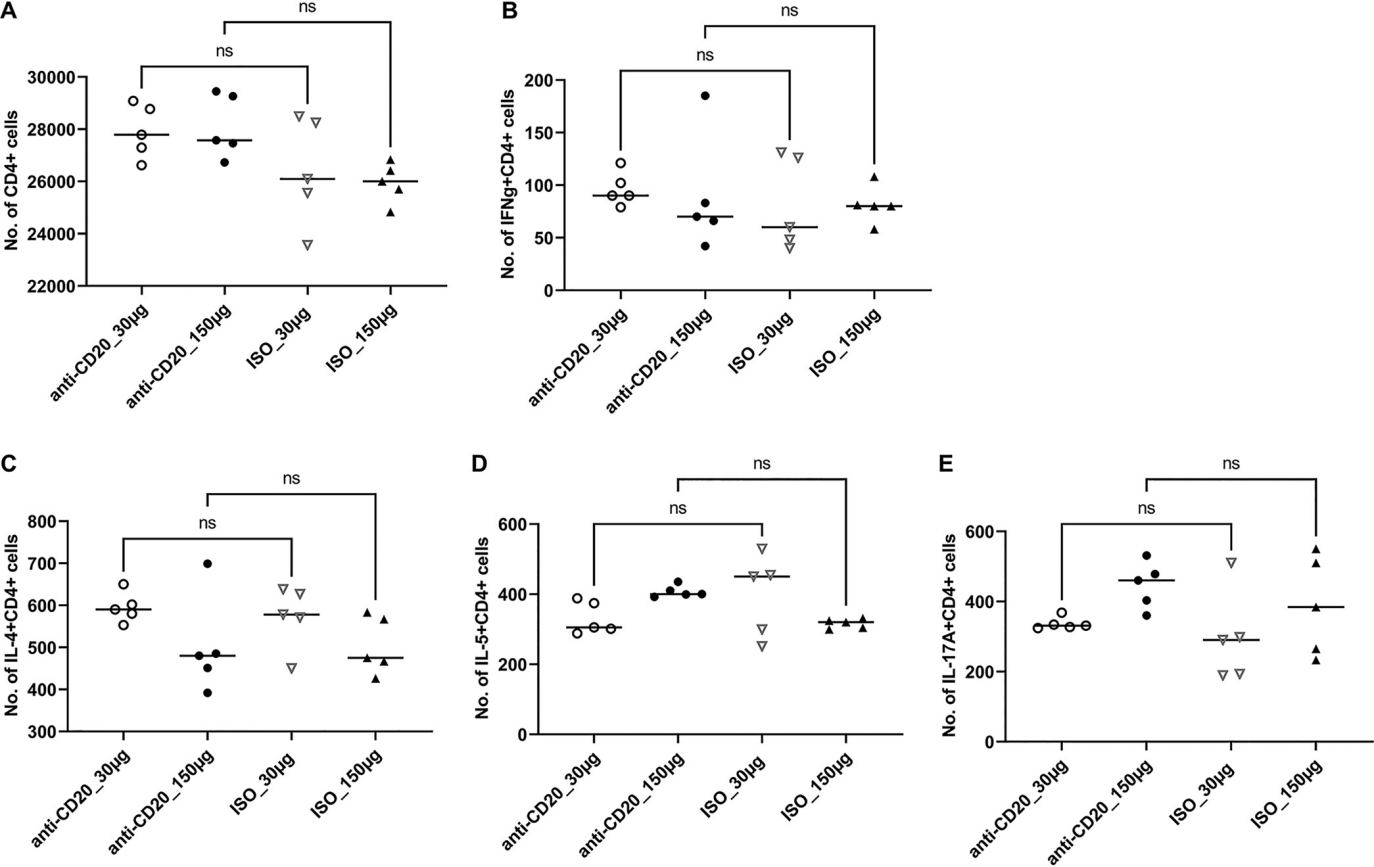
Lung CD4^+^ cells and subsets in secondary infection. All mice were inoculated with approximately 2 × 10^5^ asci of P. murina inoculum. Six weeks later, the mice were subcutaneously given 30 or 150 μg of anti-CD20 or isotype control antibody weekly. Three days after the first injection, all mice were reinoculated with approximately 2 × 10^5^ asci of P. murina inoculum. Fourteen days postinfection, the right upper and lower lung lobes were harvested, and single cells were prepared and stimulated with PC antigen for 6 h and stained for surface markers and intracellular cytokine staining. For CD4^+^ cells and subsets, at least 100,000 cells were acquired. One-way ANOVA and Tukey’s multiple-comparison tests indicated that there were no significant differences between anti-CD20- and ISO-treated groups for all CD4^+^ cell subsets. ns, not significant.

**FIG 8:**
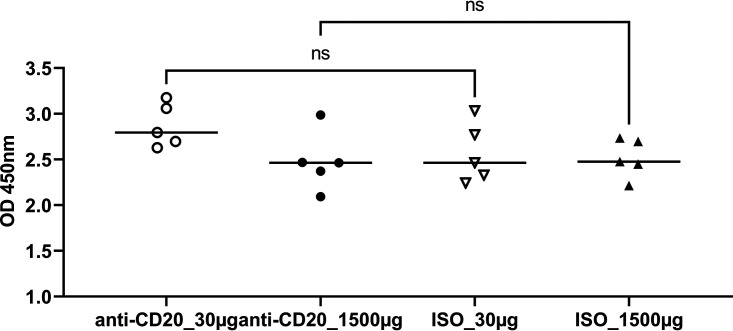
Pneumocystis-specific IgG in sera. All mice were inoculated with approximately 2 × 10^5^ asci of P. murina inoculum. Six weeks later, the mice were subcutaneously given 30 or 150 μg of anti-CD20 or isotype control antibody weekly. Three days after the first injection, all mice were reinoculated with approximately 2 × 10^5^ asci of P. murina inoculum. Fourteen days postinfection, groups of mice were sacrificed, and sera were collected for ELISA. One-way ANOVA and Tukey’s multiple-comparison tests indicated that there were no significant differences in serum IgG between anti-CD20- and ISO-treated groups. ns, not significant.

**FIG 9:**
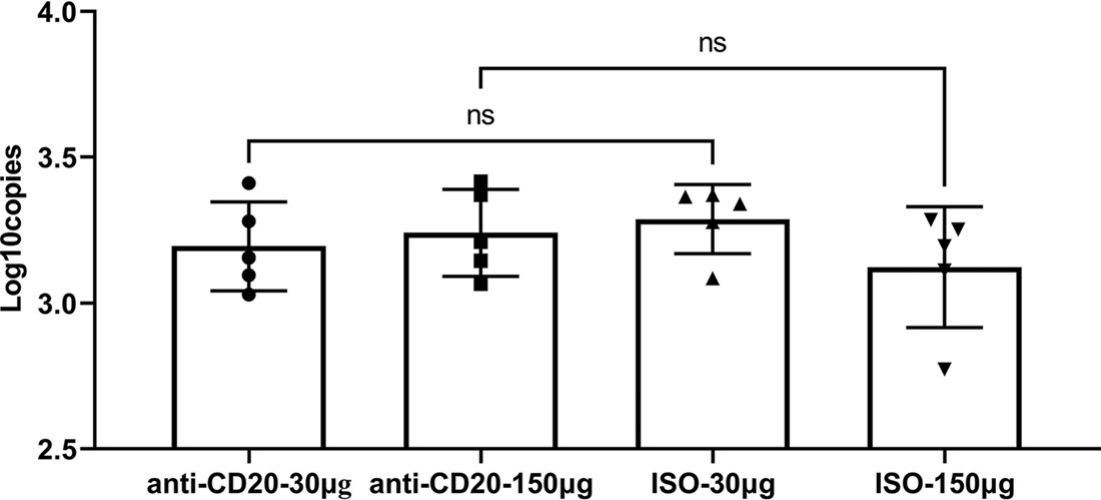
Lung fungal burden in secondary infection. All mice were inoculated with approximately 2 × 10^5^ asci of P. murina inoculum. Six weeks later, the mice were subcutaneously given 30 or 150 μg of anti-CD20 or isotype control antibody weekly. Three days after the first injection, all mice were reinoculated with approximately 2 × 10^5^ asci of P. murina inoculum. Fourteen days postinfection, the right middle lung lobes were taken, and RNA was extracted for assessing lung fungal burden by RT-qPCR. One-way ANOVA and Tukey’s multiple-comparison tests indicated that there were no significant differences in lung fungal burden between anti-CD20- and ISO-treated groups. ns, not significant.

For these studies, C57BL/6 mice were infected with P. murina and allowed to recover for 6 weeks, during which the fungi were cleared and memory B cells developed. Then mice were randomized to low- or high-dose anti-CD20 or isotype control treatment weekly. Mice were reinfected to assess the effect of anti-CD20 on preexisting humoral immunity. At 6 weeks, prior to anti-CD20 administration, there were no differences in peripheral blood B cell populations between naive mice or mice that had recovered from P. murina infection (Fig. S2). Mice were then randomized to isotype control or anti-CD20 treatment and 72 h later infected with P. murina. Fourteen days postinfection, both anti-CD20 dosing regimens resulted in reduced lung CD19^+^ cells (Fig. 6A) but not CD19/CD27^+^ cells (Fig. 6B). There were also no differences in lung CD4^+^ T cells at this time point (Fig. 7A) or in Th1 (Fig. 7B), Th2 (Fig. 7D), or Th17 (Fig. 7E) cells. There were also no differences in serum anti-Pneumocystis IgG titers between anti-CD20-treated mice and isotype controls, which were demonstrated with data obtained at 1:200 serum dilution (Fig. 8) and those at a series of serum dilutions (Fig. S2). Consistent with preexisting fungal immunity, fungal organism burdens were at the limit of detection (Fig. 9), demonstrating that anti-CD20 after prior fungal infection does not result in enhanced susceptibility to Pneumocystis infection.

10.1128/mSphere.01144-20.2 FIG S2 C57BL/6 mice were administered 100 microliters (approximately 2 × 10^5^ asci) of P. murina inoculum by oral pharyngeal aspiration (tongue pull method). Six weeks postinfection, tail blood was taken and stained for CD19^+^ and CD27^+^ CD19^+^ cells by fluorescence-activated cell sorting (FACS). The numbers of CD19^+^ and CD27^+^ CD19^+^ cells from 50,000 cells acquired were shown. Two-tailed unpaired *t* test showed that there were no significant differences of CD19^+^ cells or CD27^+^ CD19^+^ cells between groups. ns, not significant. The bottom panel shows serum IgG. C57BL/6 mice were infected with 100 microliters (approximately 2 × 10^5^ asci) of P. murina inoculum by oral pharyngeal aspiration (tongue pull method). Six weeks later, the mice were subcutaneously given 30 or 150 μg of anti-CD20 or isotype control antibody from Novartis weekly. Three days after the first injection, all mice were again infected with 100 microliters (approximately 2 × 10^5^ asci) of P. murina inoculum by oral pharyngeal aspiration (tongue pull method). Fourteen days postinfection, sera were collected, and anti-PC IgG was assayed by ELISA. There were no differences among anti-CD20- and ISO-treated groups. Download FIG S2, PDF file, 0.2 MB. Copyright © 2021 Dai et al. 2021 Dai et al. https://creativecommons.org/licenses/by/4.0/ This content is distributed under the terms of the Creative Commons Attribution 4.0 International license.

### Antibody generation by bone marrow cells.

Culture supernatants from the femurs of four Pneumocystis-infected and three control C57BL/6 mice were measured for Pneumocystis-specific IgG by using IgG mouse enzyme-linked immunosorbent assay (ELISA) kit (catalog no. 88-50400; Invitrogen). One-tailed Mann-Whitney test showed that there was a significant difference in antigen-specific IgG between control and infected groups (*P* < 0.05) (Fig. S3).

10.1128/mSphere.01144-20.3 FIG S3 Persistence of antibody generation by plasma cells. C57BL/6 mice were infected with P. murina for 6 weeks. Femurs of four infected mice and three naive controls were collected, fragmented, and cultured for 5 days in 1 ml DMEM. Media were collected and measured for pneumocystis-specific IgG by ELISA. One-tailed Mann-Whitney test showed that there was a significant difference of antigen-specific IgG between control and infected groups (*P* < 0.05). The data were shown as means ± standard errors of the means (SEM). Download FIG S3, PDF file, 0.09 MB. Copyright © 2021 Dai et al. 2021 Dai et al. https://creativecommons.org/licenses/by/4.0/ This content is distributed under the terms of the Creative Commons Attribution 4.0 International license.

## DISCUSSION

Anti-CD20 antibodies are used clinically for many diseases, including CD20^+^ malignancies as well as autoimmune diseases characterized by the generation of autoantibodies. We have previously demonstrated that parental administration of 5D2 significantly increases susceptibility to Pneumocystis pneumonia in a murine model (3). In this study, we investigated a different clone (18B12) and a subcutaneous (s.c.) route of administration that leads to partial depletion of CD20^+^ cells. At the 30-μg dose, there was a minimal impact on lung CD4^+^ T cell recruitment or the generation of an anti-Pneumocystis-specific IgG response. Within the high-dose anti-CD20 group, we observed reduced Th1 and Th2 responses with comparable higher fungal burdens postchallenge at day 14. There was considerable decrement in fungal burdens in the low-dose anti-CD20 cohort from day 14 to day 28 that was reduced in the high-dose anti-CD20 group. Thus, fungal clearance was delayed but relatively intact. In mice with preexisting immunity to Pneumocystis by prior inoculation, there was no perturbation in fungal clearance after anti-CD20 administration which was similar to our prior study with 5D2 (3). The low- and high-dose concept applied to these studies is based on the clinical setting in multiple sclerosis (MS) where anti-CD20 antibodies are used at low or high doses for treating MS patients. Ocrelizumab is given at a rather high dose of two 600-μg intravenous (i.v.) injections per year, whereas ofatumumab is given at 12 monthly 20-μg s.c. doses per year. Interestingly, the latter regimen is associated with deep and sustained B cell depletion linked to potent therapeutic effects. Clone 5D2 is a murine IgG2a antibody, whereas 18B12 is a murine IgG1 antibody. 18B12 has been investigated in reversing inhibitors (antibodies) targeting the coagulation factor VIII (FVIII) in the context of factor replacement therapy (8). Interestingly, in this study, the authors compared 18B12 with IgG2a or IgG1 backbone. The former was more effective in depleting B cells and together with rapamycin was more effective in reversing inhibitors in hemophilia A mice (8). Also, low-dose (20-μg) subcutaneous administration of the IgG2a variant reduced neuroinflammation in a model of experimental autoimmune encephalomyelitis (9). This dose and route resulted in the rapid depletion of B cells in lymph nodes, spleen, and blood. However, similar to this study, it did not reduce antigen-specific immunoglobulin M and immunoglobulin G titers after immunization. The authors attributed this to relatively intact populations of splenic marginal zone (MZ) B cells in both T cell-dependent and T cell-independent B cell immunization models (9). These data are similar to what we observed in this study with the IgG1 variant where T cell priming and clearance of fungal burden from day 14 to day 28 of infection were largely intact, particularly in the low-dose cohort. Thus, these data support the prior data that this dose and route may be helpful in mitigating diseases characterized by autoantibodies without greatly increasing risk of opportunistic fungal infections such as Pneumocystis pneumonia.

## MATERIALS AND METHODS

### Mice.

Six- to 8-week-old wild-type (WT) C57BL/6J mice were obtained from The Jackson Laboratory (Bar Harbor, ME). Immunodeficient B10:B6-Rag2^tm1Fwa^ Il2rg^tm1Wjl^ (Rag2^−/−^ Il2rγ^−/−^) mice were originally obtained from Taconic (Hudson, NY) and maintained at the Tulane University Department of Comparative Medicine (DCM) Facility. Animals were housed in a pathogen-free environment and given food and water *ad libitum*. All experiments were approved by the Tulane University Animal Care and Use Committee.

### Pneumocystis isolation, inoculum, and antigen preparation.

Pneumocystis murina organisms were administered by oral-pharyngeal delivery to *Rag2^−/−^ IL2rγ^−/−^* mice, propagated for 10 to 12 weeks *in vivo*, and isolated from mouse lung tissue as previously described (10, 11). Briefly, *Rag2^−/−^ IL2rγ^−/−^* mice with Pneumocystis pneumonia were sacrificed, and the lungs were aseptically harvested and frozen in 1 ml of sterile Dulbecco’s phosphate-buffered saline (PBS) at −80°C. To process the inoculum, frozen lungs were thawed, strained through a 70-μm filter, and pelleted by centrifugation (800 × *g*, 10 min, 4°C). The pellet was resuspended in 1 ml of PBS. A 5-μl aliquot was diluted 1:10, heat fixed on a slide, and stained with Hema-3 modified Wright-Giemsa stain (Fisher Scientific, Pittsburgh, PA), followed by ascus counting. P. murina asci were quantified microscopically, and the inoculum was adjusted to 2 × 10^6^ asci per ml. Mice were administered 100 μl (2 × 10^5^ asci) of the inoculum by oral-pharyngeal aspiration as previously described (10, 11). Pneumocystis protein antigen was prepared by differential centrifugation of the inoculum as previously described, followed by sonication of 1 mg of inoculum per ml for 5 min (3).

### Preparation of whole-lung cells (WLC).

Mice were infected with an inoculum of P. murina and then euthanized at 2 or 4 weeks postinoculation. At the time of euthanasia, mice were anesthetized by inhalation of carbon dioxide. Immediately after, mice were perfused vascularly by 5 ml of heparinized PBS injected into the right ventricle. The right superior and inferior lung lobes were then harvested, minced with razor blades, and digested in 5 ml serum-free medium with 2 mg/ml collagenase for 90 min in a 37°C shaking incubator. The cell suspension was strained through a 70-μm filter and then washed, resuspended in complete Dulbecco’s modified Eagle’s medium (DMEM) and spun, and then the cell pellet was gently vortexed and treated with ammonium chloride solution to lyse red blood cells, washed, and resuspended in 5 ml of DMEM, followed by cell counting.

### Flow cytometric analysis.

A total of 10^6^ single cells from the mouse lung were stimulated with 5 μg/ml P. murina antigen for 5 to 6 h. One hour after the start of stimulation, cells were given 1 μl/ml GolgiStop (BD Pharmingen, San Diego, CA) to block cytokine secretion. Cells were surface stained with fluorescent-conjugated antibodies for CD4 (catalog no. 100535; Biolegend), CD19 (catalog no. 152406; Biolegend), and CD27 (catalog no. 11-0271-85; eBioscience) (memory experiment only) for 30 min in PBS supplemented with 1% bovine serum albumin (BSA), washed, permeated, and stained with fluorescein-conjugated antibodies for gamma interferon (IFN-γ) (catalog no. 505850; Biolegend), interleukin 4 (IL-4) (catalog no. 504118; Biolegend), IL-5 (catalog no. 504304; Biolegend), and IL-17A (catalog no. 506916; Biolegend). Cells were acquired for flow cytometry by Cytek, and data were analyzed using FlowJo (Treestar).

### RNA isolation and Pneumocystis quantification by RT-PCR.

The right middle lobe of the lung was harvested in 1 ml of TRIzol, and homogenized. RNA was purified and quantified as previously described (10, 11). Briefly, cDNA was synthesized from 1 μg whole-lung RNA via iScript reverse transcription reagents (Bio-Rad, Hercules, CA), and real-time PCR (RT-PCR) was performed using primers and probes for the P. murina small-subunit (SSU) rRNA transcript and SsoAdvanced probe supermix (Bio-Rad). The threshold cycle values were converted to copy numbers by use of a premade standard of known Pneumocystis SSU rRNA as previously described (10, 11).

### Serum collection and P. murina antigen enzyme-linked immunosorbent assay (ELISA).

Blood samples were collected periodically by tail bleed and/or at the time of sacrifice by syringe from the vena cava. Coagulated blood samples were then centrifuged for 10 min at 10,000 × *g*. The serum supernatant was collected and stored at −80°C. Maxisorb plates were coated with 1 μg P. murina antigen in 100 μl bicarbonate coating buffer per well overnight at 4°C. Plates were blocked with 5% blotting-grade blocker (Bio-Rad) and 1% BSA. Plates were first stained with sample serum in a dilution series from 2^6^ to 2^13^ overnight at 4°C and then stained with murine Ig-specific horseradish peroxidase (HRP)-conjugated antibodies. Plates were then developed with tetramethylbenzidine (TMB) substrate for 5 to 30 min, depending on the control serum, and the reaction was stopped with an equal volume of 2 N H_2_SO_4_. The optical density at 450 nm (OD_450_) was read using a Synergy H1 Hybrid reader (BioTek, Winooski, VT).

### Antibody-mediated cell depletion.

CD20^+^ cells were depleted using anti-mouse CD20 monoclonal antibody (18B12, mouse IgG1 [mIgG1]; Novartis). An isotype control mIgG1 (anticyclosporine) was used in the control groups. Mice were subcutaneously injected with 30 μg or 150 μg antibody for different experiments. CD20^+^ cell depletion efficiency was assessed by flow cytometry using anti-CD19 antibody.

### Detection of antibody generation by bone marrow cells.

C57BL/6 mice were infected with P. murina. Six weeks after inoculation, the infection was cleared, and femurs of four infected mice and three naive controls were collected, fragmented, and cultured for 5 days in 1 ml DMEM. Media were collected and measured for Pneumocystis-specific IgG by ELISA as described above.

### Statistical analysis.

GraphPad Prism (GraphPad Software version 8.3.1, La Jolla, CA) ordinary one-way analysis of variance (ANOVA) with multiple comparisons was used to calculate *P* values.

## References

[1] Opata MM, Hollifield ML, Lund FE, Randall TD, Dunn R, Garvy BA, Feola DJ. 2015. B lymphocytes are required during the early priming of CD4+ T cells for clearance of Pneumocystis infection in mice. J Immunol 195:611–620. doi:10.4049/jimmunol.1500112. 26041535 PMC4491042

[2] Lund FE, Hollifield M, Schuer K, Lines JL, Randall TD, Garvy BA. 2006. B cells are required for generation of protective effector and memory CD4 cells in response to Pneumocystis lung infection. J Immunol 176:6147–6154. doi:10.4049/jimmunol.176.10.6147. 16670323

[3] Elsegeiny W, Eddens T, Chen K, Kolls JK. 2015. Anti-CD20 antibody therapy and susceptibility to Pneumocystis pneumonia. Infect Immun 83:2043–2052. doi:10.1128/IAI.03099-14. 25733518 PMC4399075

[4] Skalski JH, Kottom TJ, Limper AH. 2015. Pathobiology of Pneumocystis pneumonia: life cycle, cell wall, and cell signal transduction. FEMS Yeast Res 15:fov046. doi:10.1093/femsyr/fov046. 26071598

[5] Deborska-Materkowska D, Kozinska-Przybyl O, Mikaszewska-Sokolewicz M, Durlik M. 2014. Fatal late-onset Pneumocystis pneumonia after rituximab: administration for posttransplantation recurrence of focal segmental glomerulosclerosis–case report. Transplant Proc 46:2908–2911. doi:10.1016/j.transproceed.2014.09.010. 25380948

[6] Martin-Garrido I, Carmona EM, Specks U, Limper AH. 2013. Pneumocystis pneumonia in patients treated with rituximab. Chest 144:258–265. doi:10.1378/chest.12-0477. 23258406 PMC4694106

[7] Farkas JD, Clouser RD, Garrison GW. 2014. Pneumocystis pneumonia following rituximab. Chest 145:663–664. doi:10.1378/chest.13-2539. 24590034

[8] Biswas M, Rogers GL, Sherman A, Byrne BJ, Markusic DM, Jiang H, Herzog RW. 2017. Combination therapy for inhibitor reversal in haemophilia A using monoclonal anti-CD20 and rapamycin. Thromb Haemost 117:33–43. doi:10.1160/TH16-05-0404. 27683758 PMC5222884

[9] Huck C, Leppert D, Wegert V, Schmid C, Dunn R, Weckbecker G, Smith PA. 2019. Low-dose subcutaneous anti-CD20 treatment depletes disease relevant B cell subsets and attenuates neuroinflammation. J Neuroimmune Pharmacol 14:709–719. doi:10.1007/s11481-019-09872-z. 31435856

[10] Eddens T, Elsegeiny W, Ricks D, Goodwin M, Horne WT, Zheng M, Kolls JK. 2019. Transcriptomic and proteomic approaches to finding novel diagnostic and immunogenic candidates in Pneumocystis. mSphere 4:e00488-19. doi:10.1128/mSphere.00488-19. 31484742 PMC6731532

[11] Elsegeiny W, Zheng M, Eddens T, Gallo RL, Dai G, Trevejo-Nunez G, Castillo P, Kracinovsky K, Cleveland H, Horne W, Franks J, Pociask D, Pilarski M, Alcorn JF, Chen K, Kolls JK. 2018. Murine models of Pneumocystis infection recapitulate human primary immune disorders. JCI Insight 3:e91894. doi:10.1172/jci.insight.91894. PMC6124425 29925696

