# Supplementary figures and images for: Effect of Subcutaneous Anti-CD20 Antibody-Mediated B Cell Depletion on Susceptibility to Pneumocystis Infection in Mice
Source: mSphere. 2021 May 5;6(3):e01144-20. doi: 10.1128/mSphere.01144-20 (PMC8103991; doi:10.1128/mSphere.01144-20)

Fig. S1

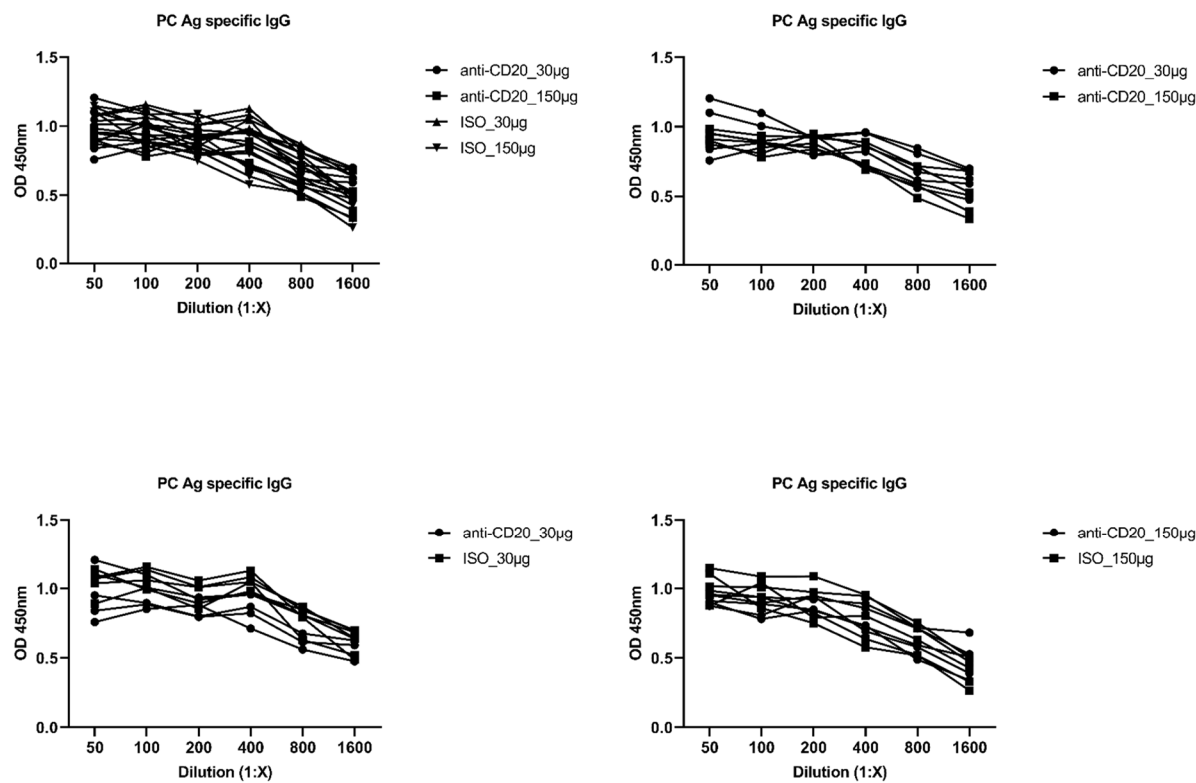

Supplement: FIG S1 [file mSphere.01144-20-sf001.pdf]

Fig. S2

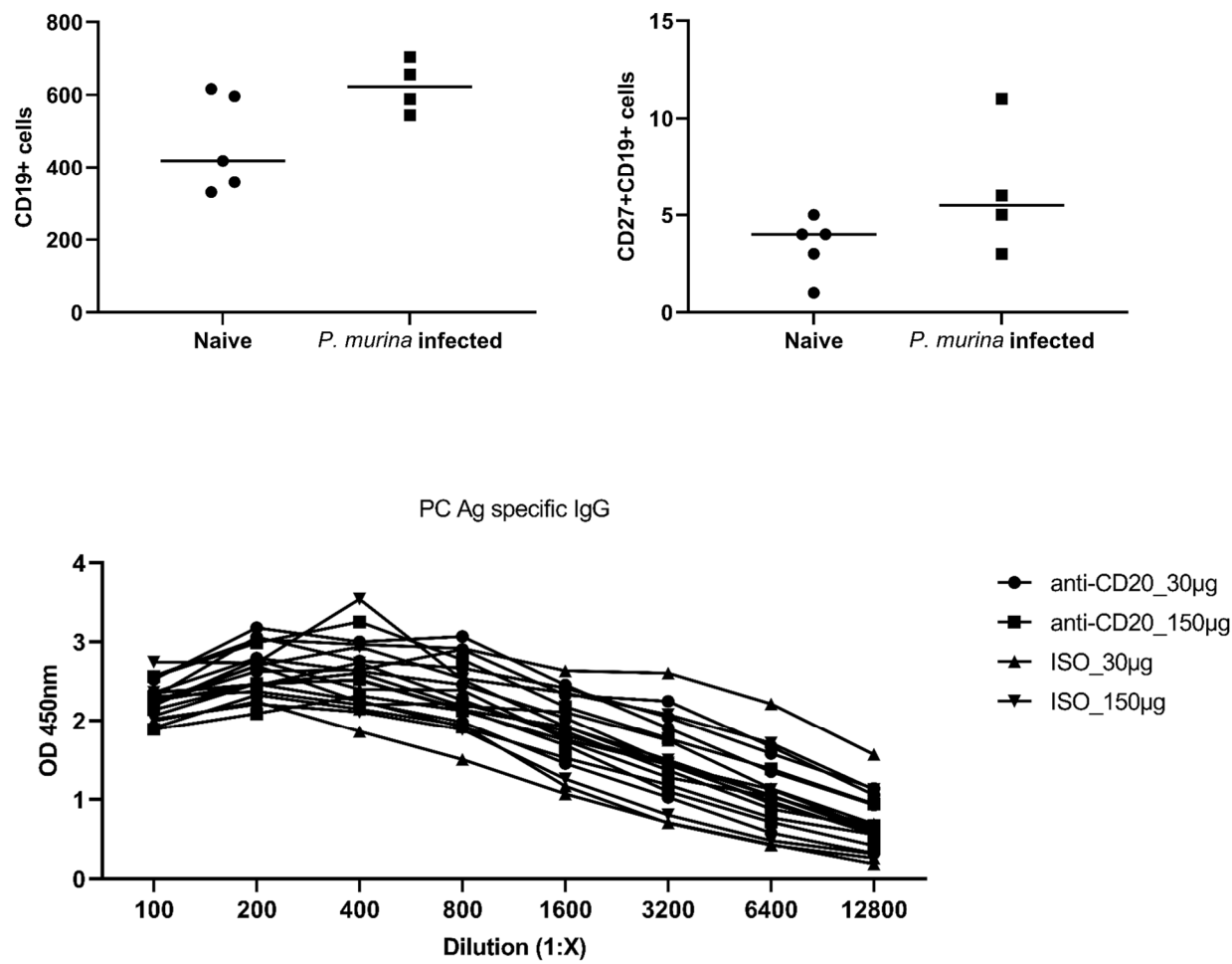

Supplement: FIG S2 [file mSphere.01144-20-sf002.pdf]

Fig. S3

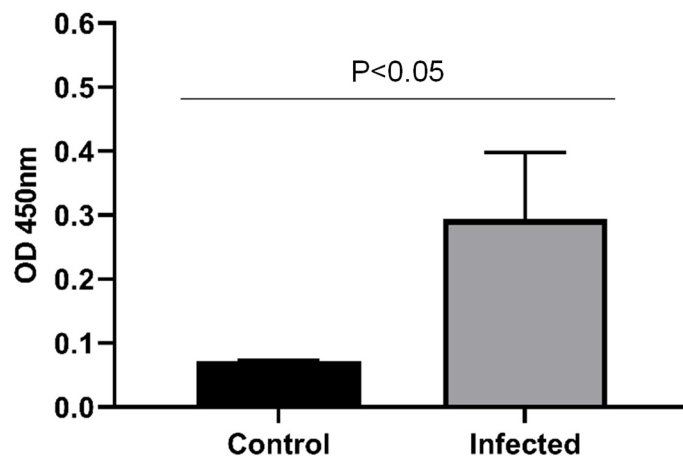

Supplement: FIG S3 [file mSphere.01144-20-sf003.pdf]
